# Tilapia Consumption and Scombroid Poisoning

**DOI:** 10.7759/cureus.5976

**Published:** 2019-10-23

**Authors:** Ehizogie Edigin, Prasanth Lingamaneni, Juan Sarmiento, Hafeez Shaka, Sanjay A Patel

**Affiliations:** 1 Internal Medicine, John H. Stroger, Jr. Hospital of Cook County, Chicago, USA; 2 Internal Medicine, OhioHealth Riverside Methodist Hospital, Columbus, USA

**Keywords:** scombroid, tilapia fish, histamine, histidine, anaphylaxis, poisoning, reportable illness

## Abstract

Scombroid poisoning, also known as histamine fish poisoning, typically occurs after eating dark meat fish. Higher levels of histidine, which is converted to histamine, causes anaphylaxis-like symptoms upon ingestion. There are few reported cases of scombroid in humans secondary to light meat fish. We present a case secondary to tilapia consumption.

## Introduction

Scombroid poisoning is the most common seafood-borne illness in the United States [1,2]. Also known as histamine fish poisoning, it is typically seen with the consumption of darker meat saltwater fish which, in comparison to lighter meat fish, contains higher levels of histidine [2-4]. Inadequate storage and refrigeration of fish allows in vivo conversion of histidine to histamine, via the enzyme histidine decarboxylase [2]. This histamine accounts for the anaphylaxis-like reaction (bronchospasm, flushing, dyspnea, hypotension, urticaria) observed with scombroid poisoning [1].

Scombroid poisoning may have a higher incidence than described due to time-limited symptoms, similarity to true anaphylaxis, and underreporting of events [2]. Acute management often necessitates a multifaceted approach, with specific awareness of airway management [4,5]. Treatment of minor symptoms (e.g., rash, flushing, nausea, cramping, diarrhea) is largely supportive. Antihistamines and steroids are often utilized. Epinephrine may be given in severe or life-threatening situations [1,3,4]. Prevention of scombroid poisoning via consumer education and public health reporting is essential.

## Case presentation

A 40-year-old woman with a history of diabetes mellitus and hypertension presented to the emergency room with a one-day onset of dyspnea and rash. She recently purchased frozen tilapia several weeks prior and stored the meat in a freezer. Two days prior to the onset of symptoms, she moved the fish from the freezer to the refrigerator. The fish was left in the refrigerator for approximately 48 hours before she prepared and consumed it. Nearly 30 minutes after eating, she noted a diffuse pruritic rash. She self-medicated with diphenhydramine with partial improvement in her symptoms. However, over the next 36 hours, she developed lip swelling, difficulty swallowing, and spread of her rash. She presented to the emergency room for worsening symptoms. Of note, her medications included captopril for the past two months and lisinopril for one year before that.

On presentation to the emergency room temperature was 98.3°F, blood pressure was 154/72 mm/Hg, heart rate was 128 beats per minute, respiratory rate was 22 cycles/minute, and she was saturating 96% on ambient air. Physical examination was significant for diffuse urticaria on her trunk and extremities. She also had bilateral hand, periorbital and lip swelling. Cardiopulmonary exam was otherwise unremarkable. Labs were notable for a leukocytosis of 13,900 cells/µL with 0% eosinophils. She was treated with intravenous diphenhydramine, famotidine, and methylprednisolone. She was also given intramuscular epinephrine. Her dyspnea and urticaria improved rapidly with treatment and she was admitted overnight for observation. By the next day, the pruritis had diminished. She no longer had dyspnea and the urticaria was receding. Her tachycardia and tachypnea had resolved and her leukocyte count decreased to 10,700 cells/µL. The patient was educated about scombroid fish poisoning and to avoid poorly refrigerated fish. She was discharged home in stable condition.

## Discussion

Histamine poisoning, commonly referred to as “scombroid fish poisoning”, results from the consumption of certain foods, typically fish and cheeses, that contain unusually high levels of histamine. Spoiled fish of the Scombridae and Scomberesocidae (e.g., tuna, mackerel, bonito) families are commonly implicated in incidents of histamine poisoning [4]. These species of dark meat fish contain higher levels of free histidine. Without appropriate storage, histidine is converted by histidine decarboxylase to histamine, causing systemic symptoms [5].

Scombrotoxic poisoning results from the improper handling and storage of fish containing naturally occurring histidine [6]. To prevent poisoning, the fish needs to be continuously stored at less than or equal to 40°F (4°C) from the time the fish is caught until it is prepared for consumption [1,2,7]. Improper handling leads to bacterial overgrowth, allowing for the conversion of histidine to histamine. Once formed, the histamine cannot be eliminated by cooking, freezing or refrigeration. Heat destroys bacteria, but already produced histamine remains unaffected due to its thermostabile properties [2].

Affected fish may not have a distinct odor or appearance which makes detection before eating difficult. Once cooked, the skin may appear honeycombed. Occasionally, patients also report a "peppery" or “spicy” taste to the fish while eating [3]. There are very few reported cases of scombroid poisoning in humans from tilapia, a lighter meat fish. Histamine levels greater than 50 mg/100 g of fish correlate with clinical toxicity [1]. In vitro public health surveillance studies have identified histamine levels as high as 290 mg/100 g of tilapia species [1,2].

Signs and symptoms appear rapidly within 30 minutes of meat ingestion. Symptoms may persist for 12 to 48 hours, of which the most commonly described are rash, flushing, headache, and diarrhea. Less common symptoms include abdominal cramps, blurred vision, dizziness, nausea, and sweating. Severe reactions cause angioedema, tongue swelling, and respiratory distress. Rarely, upper airway edema, bronchospasm or hypotension are present. Prognosis is good, with most patients demonstrating rapid improvement with the antihistamine administration. Even patients with severe features often improve within hours of treatment [3].

The mainstay of treatment for mild-moderate cases is based on the use of antihistamines (H1-blockers, H2-blockers) [8]. Corticosteroids and epinephrine may be used as adjunct therapy for severe cases, which should be managed similar to a true anaphylactic reaction [1,9].

Our patient was also receiving angiotensin coverting enzyme (ACE) inhibitor therapy which may cause similar symptoms. ACE inhibition causes bradykinin-mediated vasodilation in contrast to a histamine-mediated mechanism [10]. In this pathway, there is an absence of itching and urticaria. Their presence should suggest another etiology of symptoms [11]. Patients should be educated that this is a reaction to improper fish storage and not an allergic response to the actual fish [3].

## Conclusions

While scombroid poisoning is generally associated with dark meat fish, it can occur after ingestion of light meat fish, including tilapia. Scombroid poisoning is histamine-mediated in comparison to ACE inhibitor-induced angioedema, which is bradykinin-mediated. Rarely, scombroid poisoning can present with life-threatening symptoms. Patients with these findings warrant treatment for anaphylaxis and observation to ensure resolution of toxicity. Patients should be educated about appropriate storage and handling of meat in addition to event reporting to local public health entities.
